# Recent advances in radiotherapy of breast cancer

**DOI:** 10.1186/s13014-020-01501-x

**Published:** 2020-03-30

**Authors:** Jan Haussmann, Stefanie Corradini, Carolin Nestle-Kraemling, Edwin Bölke, Freddy Joel Djiepmo Njanang, Bálint Tamaskovics, Klaus Orth, Eugen Ruckhaeberle, Tanja Fehm, Svjetlana Mohrmann, Ioannis Simiantonakis, Wilfried Budach, Christiane Matuschek

**Affiliations:** 1 Medical Faculty, Department of Radiation Oncology, Heinrich Heine University, Dusseldorf, Germany; 2 Department of Radiation Oncology, University Hospital, LMU Munich, Munich, Germany; 3 Department of Gynecologic and Obstetrics, Evanglisches Krankenhaus Dusseldorf, Dusseldorf, Germany; 4 Department of Gynecology, Heinrich Heine University Düsseldorf, Dusseldorf, Germany

## Abstract

Radiation therapy is an integral part of the multidisciplinary management of breast cancer. Regional lymph node irradiation in younger trials seems to provide superior target coverage as well as a reduction in long-term toxicity resulting in a small benefit in the overall survival rate. For partial breast irradiation there are now two large trials available which support the role of partial breast irradiation in low risk breast cancer patients. Multiple randomized trials have established that a sequentially applied dose to the tumor bed improves local control with the cost of worse cosmetic results.

## Introduction

**Fig. 1:**
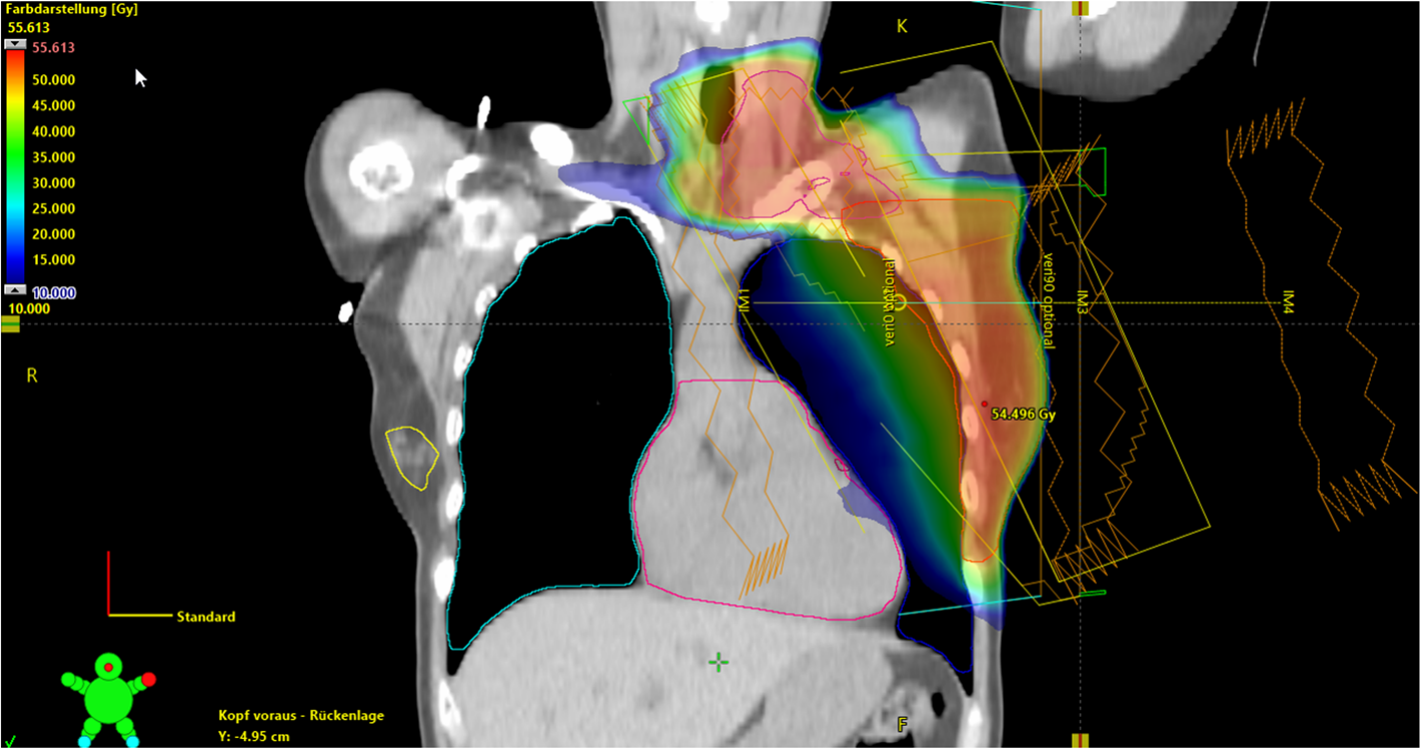
Modern treatment plan of a female patient undergoing left-sided breast and regional nodal radiotherapy using a 4-field sliding window technique. Prescription dose was 50.4 Gy in 28 fractions

Breast cancer is still the leading malignant tumor for women [1–6]. In the last few years, the treatment is improving due to new surgical techniques, new systemic therapeutic options and a better understanding of the biology of the disease [7–9]. Furthermore, there are also advances in the field of radiation oncology [10, 11] (Fig. 1). This review will report about the recent advances for treating breast cancer with radiation therapy. One aspect is the new approach regarding the radiation of regional lymph nodes especially when axillary lymph node irradiation is involved. Another important issue is the role of accelerated partial breast irradiation and the answer of the question which patients group will benefit from partial breast radiation therapy. Another aspect of this review is the answer of the questions when boost therapy should be applied. The time sequence when to perform radiation therapy is also under debate and will be discussed.

## Review

### Role of regional nodal irradiation

**Fig. 2:**
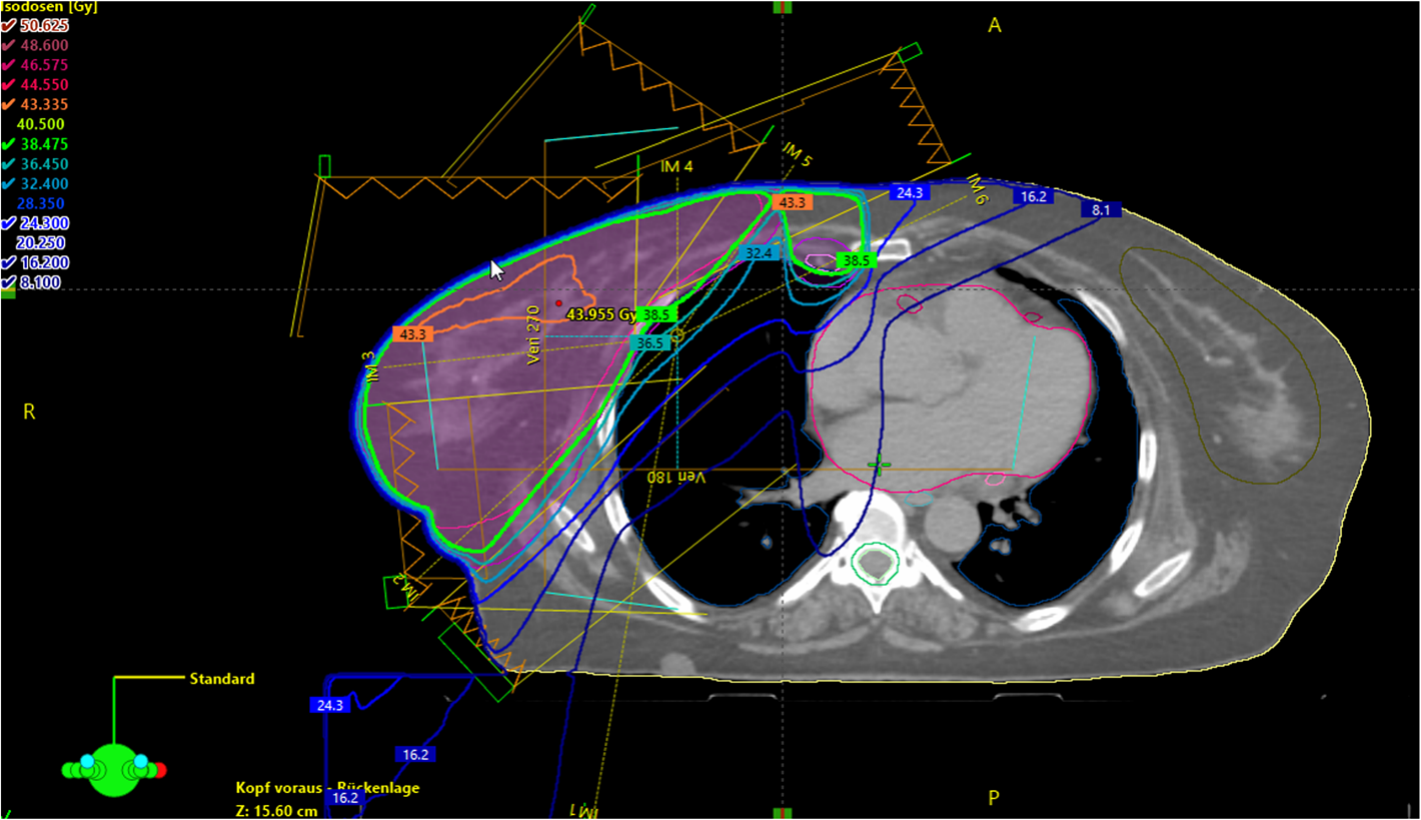
Female patient undergoing right-sided breast and regional nodal radiotherapy including supra−/infraclavicular and internal mammary lymph nodes after breast-conserving surgery using a 5-field sliding window IMRT

**Fig. 3:**
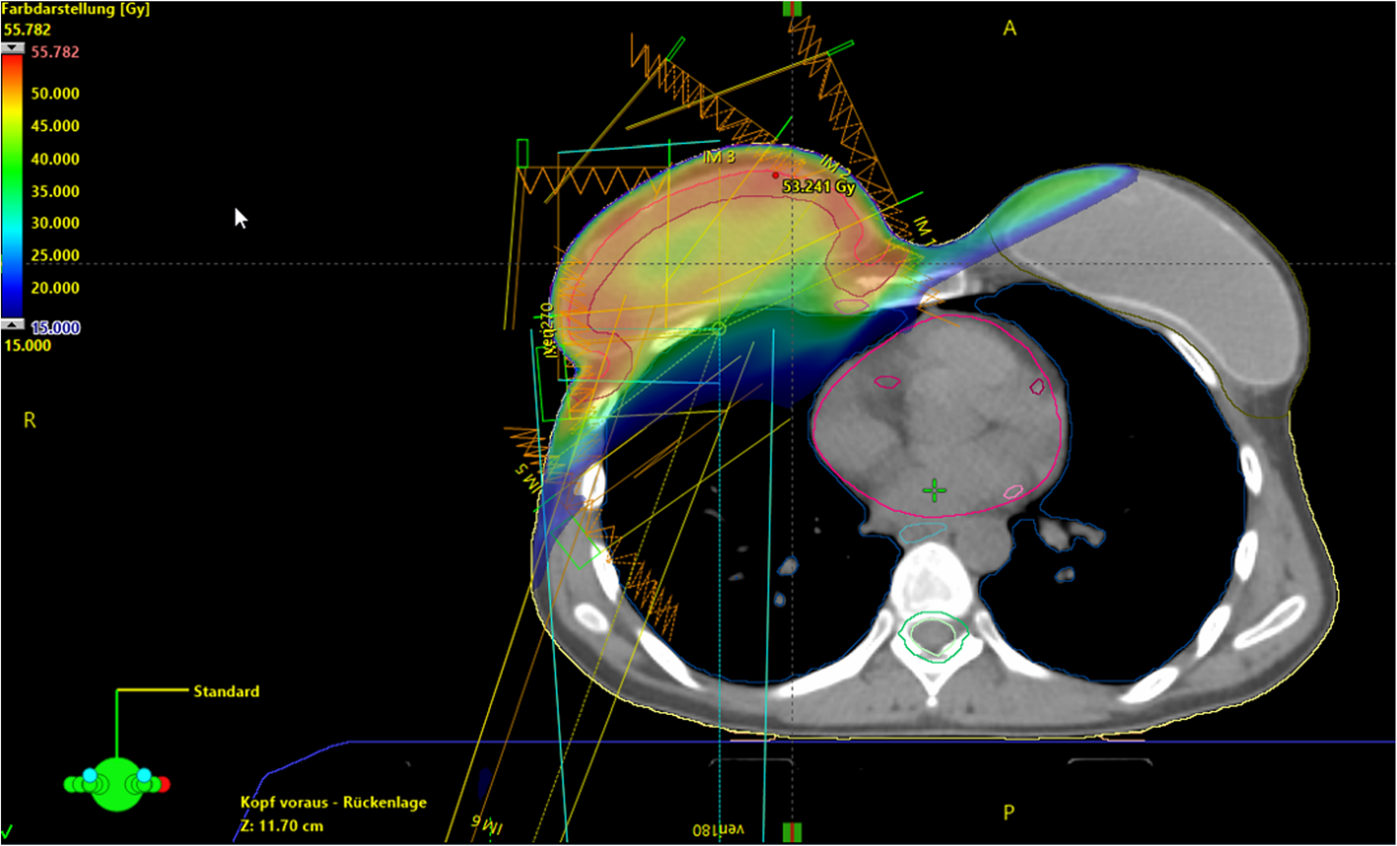
Postmastectomy patient with immediate implant-based reconstruction undergoing right sided breast and regional nodal radiotherapy including supra−/infraclavicular and internal mammary lymph nodes using a 6-field sliding window IMRT

The role of regional nodal irradiation (RNI) in breast cancer has been a matter of debate for several decades [12]. Postmastectomy radiation therapy (PMRT) implemented RNI in the adjuvant treatment of breast cancer after local resection and axillary lymph node dissection (ALND). PMRT includes the treatment of the chestwall and often the supra−/infraclavicular lymph nodes as well as the internal mammary nodes (IMN, see Figs. 2 and 3). Especially, the treatment of the IMNs remains a matter of debate, as it usually results in higher doses to heart and lungs, which have been shown to elevate the risk of non-breast cancer mortality [13]. In 2015 two large randomized trials and one prospective observational study were published which redefined the role of RNI in high-risk node negative and low volume nodal positive disease. However, due to inconsistent subgroups analyzed in the trials, physicians were unable to assess which patients had the greatest benefit of RNI. Recently, a meta-analysis of 14 trials with about 13,500 patients on individual patient data by the Early Breast Cancer Trialists’ Collaborative Group (EBCTCG) was presented at the San Antonio Breast Cancer Symposium (SABCS) in December 2018 [14]. Adding RNI to chest wall or breast irradiation showed an improvement in any recurrences (− 2.9%) and breast cancer death (− 4.0%). The investigators further divided the available data according to the recruitment date (1961–1978 and 1989-onwards). Both groups achieved a median follow-up of 9.2 years. They showed that the older trials usually treated patients with outdated RT techniques and reached mean heart doses of > 8 Gy, whereas the newer studies had a lower heart exposure. When analyzed separately, RNI reduced the appearance of any recurrence (10-year gain: 3.2, 95% CI: 1.3–5.1%) in the newer trials, whereas the older trials did not show any effect. The same effect was seen likewise in breast cancer mortality (10-year gain: 2.8, 95% CI: 1.2–4.4%) and overall survival (10-year gain: 2.9, 95% CI: 1.2–4.6%). On the contrary, RNI was even associated with a loss in 20-year overall survival (− 2.8, 95% CI: − 2.1-7.7%) in the older trials. These differences are explained by a higher rate of non-breast-cancer mortality (20-year: 5.8, 95% CI: − 1.1–12.7%), while in the newer studies this endpoint was not statistically significant. Taken together, newer RNI techniques appear to provide better target coverage and a reduction in long-term toxicity, resulting in a small, consistent benefit in overall survival.

In terms of subgroup that might benefit most from RNI, the analysis showed that the relative benefit was the same in all nodal subgroups (0, 1–3 or 4+ positive nodes). However, the absolute benefit of RNI in breast cancer mortality was the largest in the subgroup of patients with > 4 positive lymph nodes. When analyzing the value of RNI according to the location of the primary tumor, the authors reported that medial or central tumors had a numerically greater mortality reduction. Further, patients receiving an endocrine or systemic therapy appeared to derive more benefit of RNI compared to no systemic therapy. Additionally, the inclusion of all relevant lymphatic drainage sites in the radiation volumes seemed to results in the best therapeutic effect. When putting these data into current clinical practice it should be noted that a majority of patients in these trials received complete axillary nodal dissection and adjuvant systemic therapy only. Currently, many patients are treated with neoadjuvant chemotherapy followed by an evaluation of the nodal status using sentinel node biopsy. This sequence underestimates the nodal burden compared to a resection first approach which complicates the appropriate indication for RNI as the evidence was generated with the knowledge of the full extend of the patient’s disease. Currently it is unknown whether the pre-therapeutic or the postoperative status should be evaluated when assessing the indication for RNI. Further randomized studies are ongoing in this context.

In summary, these data provide strong evidence that RNI including the IMN should be considered in patients with high volume nodal disease or high-risk N0–1 disease using modern radiation techniques.

### Axillary radiotherapy

At SABCS 2018, Emil Rutgers presented the updated results of the EORTC AMAROS trial with a follow-up period of 10 years [15]. In this trial, patients with a cT1–2 tumor and clinical nodal negative disease were randomized between axillary lymph node dissection (ALND) and axillary radiotherapy (AxRT). Only in case of a positive sentinel lymph node (SNLB+) patients were treated within the trial protocol. Neoadjuvant systemic therapy was not allowed. The AxRT started within 12 weeks after the sentinel lymph node biopsy (SNB) and included axillary lymph node levels I-III and the medial supraclavicular level. The included patients received breast-conserving surgery (~ 82%) or mastectomy (~ 18%), as well as systemic treatment with adjuvant chemotherapy (~ 60%) and endocrine therapy (~ 78%). Overall, most patients received RT to the breast or the chest wall (~ 85%). In the ALND arm, 32.8% of patients had additional positive lymph nodes following ALND.

After 10 years follow-up, the ALND arm reported 7 events compared to 11 events in the AxRT arm, resulting in a hazard ratio of 1.71 (CI95%: 0,67–4.39; *p* = 0.365), demonstrating no significant difference between the two treatment arms. The analysis of the overall survival similarly showed no difference between the two arms (HR: 1.17; CI-95%: 0.89–1.52). Regarding side effects, the endpoint lymphedema by clinical observation and/or treatment after 5 years was significantly lower after AxRT as compared to ALND (14.6% versus 29.4%, *p* < 0.0001).

The results of this trial confirm the trial of the previously published OTOASOR trial [16]. This trial compared the completion of axillary lymph node dissection (cALND) to regional nodal irradiation (RNI) in patients with sentinel lymph node metastasis (pN1sn) in stage I-II breast cancer. The long term follow-up results of this prospective-randomized trial suggested that RNI without cALND does not increase the risk of axillary failure in selected patients with early-stage invasive breast cancer (cT ≤ 3 cm, cN0) and pN1(sn).

The ACOSOG Z0011 [17] trial tested the omission of complete ALND in clinically lymph node negative and SNB positive patients. This trial showed no significant differences between the groups. RT in this trial was restricted to tangential breast treatment. However, when the treatment fields where analyzed, the majority of patients received substantial doses to the axillary lymph nodes which might have contributed to the favorable outcome [18].

Overall, together with the data from the ACOSOG Z0011 [17] and the OTOASOR trial [16], these long- term results confirm the role of axillary radiotherapy as a valid alternative to ALND in the management of primarily surgically managed patients with clinically negative and SNB positive tumors, with significantly less morbidity. In contrast to the Z0011 trial, the AMAROS trial also included patients following mastectomy.

### Partial breast irradiation (PBI)

**Fig. 4:**
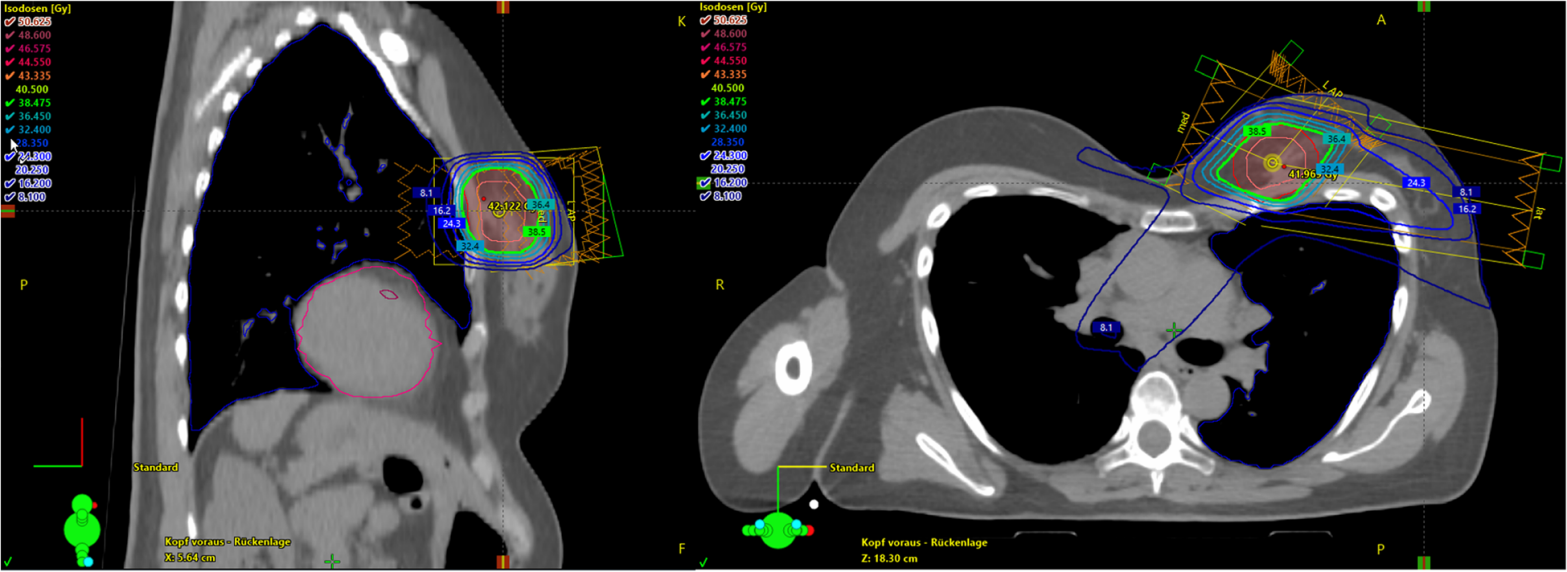
Female patient undergoing left-sided partial breast radiotherapy using a 3-field sliding window IMRT technique for a low risk tumor. Moreover, the patient had a pectus excavatum

The observation that most in-breast recurrences are located at the original tumor bed lead to clinical comparing whole breast irradiation (WBI) and partial breast irradiation (PBI) [19]. An example is given in Fig. 4. PBI was thought to be an attractive alternative that might reduce doses to the organs at risk (OARs) and could also provide a better cosmetic results in a shorter treatment time than 3–6 weeks. There are multiple technical approaches to irradiate a partial volume of the breast in comparison. In the completed trials, APBI was delivered using intraoperative radiation, brachytherapy or external beam techniques [20–24].Local recurrence rates were overall slightly higher in the partial breast groups, especially in patients with higher risk breast cancer subtypes [20, 22, 25]. Results of two large randomized trials (NSABP B-39/RTOG 0413, RAPID) were recently presented at the SABCS18 and subsequently published in the Lancet in 2019 [26, 27].

The RAPID trial randomized patients between whole breast irradiation using a regimen of 50 Gy in 25 fractions or 42.5 Gy in 16 fractions with an optional boost of 10 Gy in 4–5 fractions to the tumor bed or APBI using a 3D conformal (3D-CRT) or intensity modulated external beam radiation (IMRT) technique with 38.5 Gy in 10 twice daily fractions. The primary objective was to test non-inferiority regarding ipsilateral breast tumor recurrences (IBTR) of APBI compared to WBI. The trial included patients older than 40 years with DCIS or invasive carcinoma or smaller than 3 cm, with clear resection margin and node negative disease. Lobular histology and multi-centric disease were exclusion criteria. After a median follow-up of 8.6 years, the results of 1070 patients in the APBI arm were compared to 1065 patients in the WBI arm. The median age was 61 years, and 82% of patients presented with invasive disease, a small tumor size < 1.5 cm (69%) and ER positivity (84%). Chemotherapy was utilized in ~ 15% of participants.

The analysis of the primary endpoint of IBTR showed 37 events (3.0%) in the APBI arm and 28 events (2.8%) in the WBI arm, resulting in a hazard ratio of 1.27 (90%-CI: 0.84–1.91), which was below the prespecified non-inferiority margin upper bound of 2.02. Disease-free survival, event-free survival and overall survival also showed no differences between treatments. The authors subsequently also reported radiation-associated acute and late toxicity. Acute grade ≥ II° toxicity was more prevalent (28% vs. 47%) in the WBI arm. However, late toxicity grade ≥ II° was more common after ABPI (32% vs. 13%). Furthermore, cosmesis rated as fair/poor compared to excellent/good by nurses (36% vs. 19%) or patients (31% vs. 15%) was more prevalent in the APBI arm at year 7. This fact might be influenced by the prescription dose (38.5 Gy in 10 fractions), the fractionation scheme (twice daily with 6–8 h interval) and the relatively large PTVs used in the study. These detrimental effects of APBI on cosmesis were unexpected and are subject to further analysis.

Additionally, another large randomized multicenter trial addressing ABPI was presented at SABCS18. The NSABP B-39/RTOG 0413 trial randomized patients with DCIS or stage I-II breast cancer to whole breast radiotherapy using fractions of 1.8–2.0 Gy to 50–50.4 Gy with on optional boost to ≥60 Gy or ABPI. In this study, different APBI techniques including brachytherapy and 3D conformal external beam technique were allowed. Overall 4216 patients with a median age of 54 years were included. 24% had the diagnosis of a DCIS and 27% received adjuvant chemotherapy. 71% were treated with a 3D conformal RT consisting of 10 fractions of 3.85 Gy in 5–8 days. The primary endpoint was also IBTR with the aim to test equivalence of ABPI after a median follow-up of 10.2 years. The primary endpoint analysis of IBTR showed 90 events (4.6%) in the ABPI arm and 71 events (3.9%) in the WBI arm, resulting in a hazard ratio of 1.22 (90%-CI: 0.94–1.58). This did not meet the prespecified non-inferiority margin upper bound of 1.5. Distant-, disease-free and overall survivals were not different between the groups. A subgroup analysis of IBRT revealed that the recurrences in the APBI arm occurred with the same frequency at the site of the tumor bed as in the WBI arm. However, recurrences were more likely to occur elsewhere in the breast in the APBI cohort (1.5% vs. 2.7% HR = 1.99 CI-95%:1.23–3-23). An exploratory analysis of the APBI methods suggested that the used brachytherapy methods were associated with higher recurrences rates (7.7% multi-catheter brachytherapy and 7.8% single-entry brachytherapy compared to EBRT APBI (3.7%) and WBI (3.8%). Adverse events as well as the incidence of second cancers were not different.

Additionally, quality of life (QoL) and cosmetic results in a substudy were reported at ASCO and ASTRO 2019. Here the PBI patients reported less fatigue, breast pain and treatment-related symptoms in the immediate post treatment period. Patient rated cosmetic outcome based on the global health status was equivalent for PBI and WBI after 36 months (fair/poor: PBI 29% vs. WBI 20%). Accruing site MDs rated cosmetic outcome worse at 36 months for PBI (fair/poor: PBI 26% vs. WBI 14%). The authors also demonstrated that patients’ satisfaction as equal in both groups.

In conclusion, these two large studies contribute fundamentally to the question of which target volume of the breast should be irradiated. Both studies are the first large-scale trials to indicate the efficacy endpoint after a median follow-up of more than 8 years. Additionally both trials reported multiple prespecified and exploratory subgroups analyses that did not show a difference between partial and whole breast radiation in these subgroups. A meta-analysis of previous trials reported a slightly higher rate of local recurrences. This was possibly attributed to the inclusion of patients with a higher clinical or biological recurrence risk [20, 21]. However when analyzing the single trial data only the Italian IORT trial had a significantly higher local recurrence rate [20].

Overall, PBI appears to be an acceptable alternative to WBI when selecting for the appropriate risk group and treatment modality while we await a comprehensive meta-analysis of the trials on individual patient data.

PBI is an acceptable alternative when using interstitial brachytherapy, intensity modulated radiotherapy with 5 × 6 Gy over 2 weeks or 3 D conformal radiotherapy (15 × 2,67 Gy) over 3 weeks in low risk patients (pT1,pN0, R0, G1–2, HR+, non lobular histology, age > 50 years, no extensive DCIS component).

### Hypofractionated post-mastectomy radiotherapy

Multiple randomized trials compared normofractionated (25–28 Fractions in 1.8–2.0 Gy single doses) to hypofractionated RT schedules (single doses > 2.0 Gy in 15–16 fractions) [28, 29]. Overall, fraction sizes greater than 2.0 Gy did not affect local recurrence, was associated with decreased acute toxicity and did not affect breast cosmesis, late toxicity or quality-of-life [30, 31]. These shorter regimens have been adopted mainly for lower risk patients after breast conserving surgery (BCS) but not thoroughly in PMRT with treatment of the RNI due to concerns regarding risk of lymphedema and brachial plexopathy. This is despite some data with older techniques and longer follow-up of patients within the British Columbia trial. High-risk patients after mastectomy and chemotherapy were randomized to 35–37.5 Gy in 16 fractions of PMRT including RNI or no further therapy [32, 33]. This trial showed an improvement in overall survival (37 to 47%) with a slight increase in lymphedema rate and no cases of brachial plexopathy. In the British START trials overall 864 patients received RNI and a pooled analysis of those patients showed no effect of fraction sizes on side effects including lymphedema or difficulty raising the arm [28, 34].

Recently, a large Chinese phase III trial was published in the Lancet Oncology [35]. In this study, 820 patients aged 18–75 years with N2 or T3–4 disease were randomized to receive either 50 Gy in 25 fractions or 43.5 Gy in 15 fractions. At a median follow-up time of about 5 years the locoregional recurrence rate 8.3% and 8·1% (HR = 1.10 90%-CI: 0.72 to 1.69). There were also no differences in overall survival or late toxicities. However, patients in the hypofractionated radiotherapy group had higher grade 3 acute skin toxicity (8% vs. 3%) [35].

In summary, the results of the published trials as well as multiple ongoing trials will provide sufficient information for the question whether hypofractionation is oncological equivalent and safe in the setting of PMRT.

### Boost therapy

Several randomized trials have established that a sequentially application of a localized dose escalation (boost) to the tumor bed improves local control but at the expense of poorer cosmetic results [36–40]. The use of a simultaneously integrated boost (SIB) during the whole breast treatment has several theoretical dosimetric advantages, as the dose can be reduced for the remaining breast as well as for organs at risk (OAR).

The multicenter randomized IMPORT HIGH study aimed to compare a simultaneously or sequentially applied boost regarding the endpoints of local relapse and normal tissue effects. At the SABCS18 the results of the patient and clinician reported outcomes of the normal tissue effects were reported [41]. The IMPORT HIGH trial included 2617 patients that underwent breast-conserving surgery with primary tumors stages pT1–3, pN0-3a, cM0 and were randomized between three groups. The standard arm received 15 fractions of 2.7 Gy to 40 Gy to the whole breast with a boost of 8 fractions of 2 Gy. The two experimental arms were treated with either: (i) 15 fractions of 2.4 Gy to 36 Gy to the whole breast, 2.7 Gy to 40Gy to the tumor bed with an additional margin and 3.2 Gy boost to the tumor bed to 48Gy (48 Gy group) or (ii) the same treatment with a dose escalation of the boost of 3.5 Gy to 53 Gy to the tumor bed (53Gy group).

The rates of `quite a bit or very much´ breast induration after 39 months were 13.6, 13.4 and 17.5% in the 40 Gy−/48 Gy−/53 Gy-groups, respectively. The differences between 48 Gy and 53 Gy were statistically different, whereas the other patient and clinician reported outcomes were not different statistically significant between the two boost groups. This report is the largest and most sophisticated regarding adverse effects of a SIB treatment within a prospective trial. Both SIB regimens showed a comparable side effect ratio in relation to the control arm. Further, the trial showed a dose response for adverse events in the two experimental arms. The SIB concept in higher risk breast cancer appears to be an attractive possibility in order to shorten treatment time with comparable side effects. However, clinical efficacy has not been determined yet.

In this context the forthcoming data of the Hyposib study will be important. This trial tested the sequential or the simultaneous application of a tumor bed boost in the context of a hypofractionated treatment of the whole breast. The experimental group received hypofractionated radiotherapy of the breast 16 × 2.50 Gy with simultaneous integrated boost to the tumor bed, total dose within the boost volume 16 × 3.00 Gy. The primary endpoint measure of this trial is progression-free survival [42].

### Modern radiotherapy techniques

#### Heart sparing techniques in breast radiotherapy

Over the last decade, the awareness for cardiac morbidity during breast cancer radiotherapy has increased significantly. The population-based case-control study of Darby et al. [43] analysed 2168 patients, who underwent radiotherapy for breast cancer between 1958 to 2001 in Sweden and Denmark, regarding the risk for major coronary events. The study revealed that the excess relative risk of major coronary events increased linearly with the mean heart dose by 7.4% per Gray (95% confidence interval, 2.9 to 14.5; *P* < 0.001). But we have to keep in mind this corresponds to an increase in the relative risk of 1.074 per Gy. If we look at the absolute value with a 10% base event rate without RT we will find an increase to 10.74% for 1 Gy mean heart dose. Therefore, it is of highest interest to keep the heart dose exposure as low as possible during left-sided breast RT. A recent literature review by the German society of radiation oncology (DEGRO) [44] recommended a dose constraint of < 2.5Gy for the mean heart dose for breast RT without RNI.

Furthermore an increased mortality rate from cardiac events was generally no longer observed in patients irradiated after around 1980. If only the breast is irradiated, the effect in the Darby publication may be overestimated.

Especially in cases of left-sided IMC irradiation, increased rates of cardiovascular disease (CVD) and ischaemic heart disease (IHD) may be expected. Nevertheless, in the EBCTCG meta-analysis analyzing the impact of RNI, studies from 1989 to 2003 had a lower heart exposure than older studies (1961–78) and did not increase non-breast cancer mortality.

Overall, it remains unclear if the mean heart dose is the optimal parameter to predict the risk of cardiac events. Nevertheless, in modern breast RT, the mean heart dose can be kept at reasonable levels [45], even if subvolumes of the heart (e.g. heart apex) and the left anterior descending artery (LAD) can still be exposed to much higher doses. Several RT techniques have lately been developed to spare dose exposure of the heart. Modern intensity modulated (IMRT) or volumetric modulated (VMAT) RT techniques can be used to shape the dose distributions around the heart in order to reduce the high-dose volume [46]. Nevertheless, this is usually at the cost of a low-dose spill to the lungs, the contralateral breast and the whole heart [47]. Therefore, the application of these techniques might be associated with an increased risk of radiation-induced secondary lung cancer, especially in smoking patients [48]. Another option is APBI, as discussed above. Due to the smaller target volumes in patients receiving APBI, the dose exposure of the heart might be lower as compared to whole breast irradiation. A meta-analysis actually shows a slightly lower non-breast cancer mortality according to APBI compared to WBI [49]. With the addition of the recently published trials the difference is no longer statistically significant (Haussmann et al. ESTRO poster).

Another promising technique for breast only RT is prone-positioned breast irradiation, especially in patients with large breasts [50]. This technique exploits gravity to elongate the treated breast away from the heart and lung. Whole breast irradiation in prone position has been shown to reduce mean heart and ipsilateral lung dose exposure [51]. However, prone RT can only be used when no RNI is planned where the heart dose is usually already quite low.

**Fig. 5:**
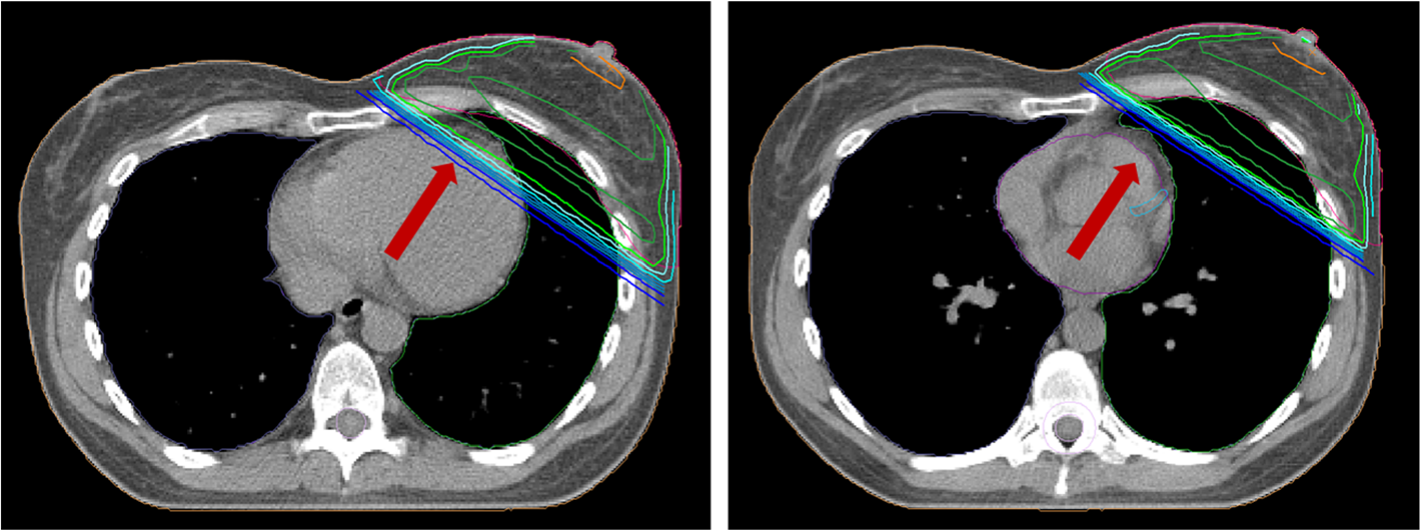
Anatomic position of the breast target volume and heart in free-breathing (left picture) and deep inspiration breath-hold (right picture)

Deep inspiration breath hold (DIBH) RT mechanically increases the distance of the heart from the chest wall and can therefore spare the heart (see Fig. 5). During DIBH, the patient inhales to a specified level and holds his breath during image acquisition and treatment delivery. Different technical solutions are used for DIBH RT, as voluntary or computer-controlled DIBH techniques [52]. Usually these can be divided into surface-guided or spirometry-based systems [53]. DIBH is compatible with both, 3DCRT and IMRT/VMAT techniques [54], and also with APBI techniques [55]. Overall, DIBH results in considerably lower doses to the apex of the heart and other cardiac substructures (e.g. LAD). Although all breast cancer patients might benefit from DIBH in treatment planning delivery, highest benefits are expected for left-sided breast cancer patients with a favourable tumour prognosis, high mean heart dose or high baseline IHD risk, independent of their age [3]. If only SC is irradiated, the mean heart dose is also significantly reduced. At IMC RT, it depends on the individual case.

Of note, DIBH seems to have an impact on the unintended regional nodal irradiation during tangential field breast radiotherapy. A significant dose variability and movement of the axillary lymph node levels is observed during DIBH in the anterior and cranial directions. This movement leads to a significant dose reduction in level I [56], and also in level II, in case of 3DCRT locoregional breast irradiation [57]. Nevertheless, to date, the potential relevance of a decreased unintended regional nodal irradiation in the era of deescalated axillary dissection or following neoadjuvant chemotherapy regimens remains under investigation. However, the AGO guideline recommends a RT of level I-II to 0.5 cm below the axillary vein if 1–2 SN are affected in the axilla in the case that no axilla dissection was performed.

### Neoadjuvant radiochemotherapy

The time sequence when to perform radiation therapy is still a question of debate and a large prospective multicenter trial in Germany will attempt to answer this question. Matuschek et al. have shown in a single institute analysis that neoadjuvant radiochemotherapy for advanced breast cancer is a safe and reliable method of treating these patients [58]. They analyzed the cosmetic results of 315 patients with neoadjuvant radiochemotherapy in a long term follow up. In their investigation, they were able to show that the cosmetic results as well as the quality of life were favorable. 80% of all breast conserving surgery patients evaluated their overall cosmetic result as “excellent” or “good” as compared to 55.8% after mastectomy patients. Patient and panel ratings on cosmetic outcomes were similar between the two groups. No grade III or IV fibrosis were identified in any of these patient groups. The median breast retraction score after breast conserving surgery was 2.9 [58]. Further, quality of life (QoL) also seemed to not inferior to the general age-matched population [59]. The strength of the study was the very long follow-up of 14–21 years (mean 17.7). However, given the retrospective nature of the trial and its inherent selection bias prospective data are required to evaluate this approach.

## Conclusion

Radiation therapy of patients with breast cancer remains a challenging and rapidly changing treatment. Regional lymph node irradiation in younger trials appears to deliver superior target coverage as well as a reduction in long-term toxicity subsequent leading to a small benefit in the overall survival rate. For partial breast irradiation there are now two large trials published which support the role of partial breast irradiation for low risk breast cancer patients. Multiple randomized trials have established that a sequentially applied to boost the tumor bed improves local control with the cost of worse cosmetic results but simultaneously integrated boost might provide equal results in terms of local control as well as adverse events. Neoadjuvant radio chemotherapy is still considered as a risk factor for impaired wound healing and risk factor for a poor cosmetic outcome, but recent publications seems to change this attitude. A prospective randomized trial will be established in Germany to answer this question.

## Data Availability

Not applicable.

## Abbreviations

ALND: Axillary lymph node dissection
APBI: Accelerated partial breast irradiation
ASCO: American Society of Clinical Oncology
ASTRO: American Society for Radiation Oncology
CI: Confidence interval
DCIS: Ductal carcinoma in situ
DEGRO: German society of radiation oncology, Deutsche Gesellschaft für Radioonkologie
DIBH: Deep inspiration breath hold
EBCTCG: Early Breast Cancer Trialists’ Collaborative Group
EBRT: External beam radiation therapy
ER: Estrogen receptor
GTV: Gross tumor volume
Gy: Gray
HR: Hazard ratio
IBTR: Ipsilateral breast tumor recurrence
IMC: Internal mammary chain
IMRT: Intensity modulated radiotherapy
LAD: Left anterior descending artery
MD: Medical Doctor
MR: Magnetic resonance imaging
NA-PBI: Neoadjuvant partial breast irradiation
OAR: Organs at risk
PBI: Partial breast irradiation
PMRT: Postmastectomy radiation therapy
PTV: Planned target volume
Qol: Quality of life
RNI: Regional node irradiation
RT: Radiotherapy
SABCS: San Antonio Breast Cancer Symposium
SIB: Simultaneously integrated boost
VS: Versus
WBI: Whole breast irradiation
